# Social vulnerability index associated with higher COVID-19 seroprevalence in Nigeria

**DOI:** 10.1080/16549716.2024.2446043

**Published:** 2025-01-21

**Authors:** Natalia Blanco, Olanrewaju Lawal, Jibreel Jumare, Christina Riley, James Onyemata, Thomas Kono, Anna Winters, Chenfeng Xiong, Alash’le Abimiku, Manhattan Charurat, Kristen A. Stafford, for The INFORM Africa Research Study Group

**Affiliations:** a Institute of Human Virology, University of Maryland School of Medicine, Baltimore, Maryland, USA; b Department of Geography and Environmental Management, Faculty of Social Sciences University of Port Harcourt, Port Harcourt, Nigeria; c Akros, Lusaka, Zambia; d International Research Center of Excellence, Institute of Human Virology Nigeria, Abuja, Nigeria; e Minnesota Supercomputing Institute, University of Minnesota, Minneapolis, Minnesota, USA; f Department of Civil and Environmental Engineering, College of Engineering, Villanova University, Villanova, PA, USA; g Department of Medicine, University of Maryland, School of Medicine, Baltimore, Maryland, USA; h Department of Epidemiology and Public Health, University of Maryland School of Medicine, Baltimore, Maryland, USA

**Keywords:** Social vulnerability index, Nigeria, COVID-19, seroprevalence, social determinants

## Abstract

Social vulnerability has been shown to be a strong predictor of disparities in health outcomes. A common approach to estimating social vulnerability is using a composite index, such as the social vulnerability index (SVI), which combines multiple factors corresponding to key social determinants of health. Lawal and Osayomi created an SVI to explore key social determinants of health-related COVID-19 infection among the Nigerian population. This study explored the association of COVID-19 SVI with COVID-19 seroprevalence using a large household survey in Nigeria. Weighted COVID-19 seroprevalence estimates at the Local Government Areas (LGA) were estimated and merged with the Lawal and Osayomi SVI, also at the LGA-level. Linear regression models were constructed to evaluate the relationship between the SVI and COVID-19 seroprevalence. The effect of SVI was evaluated both as a continuous variable and categorized into quintiles to evaluate dose–response effects. Our results confirmed a positive relationship between social vulnerability and COVID-19 infection in four states and the Federal Capital Territory in Nigeria. Compared to class 1 (the least vulnerable group), COVID-19 seroprevalence was, on average, 9.21% and 6.42% higher in classes 4 and 5 LGAs, respectively, after adjustment by phase of the survey. The effect was particularly strong farther into the pandemic (June 2021), when COVID-19 mitigation measures were relaxed. In conclusion, SVI can potentially be a useful tool to effectively prioritize communities for resource allocation as part of emergency response and preparedness in Africa.

## Paper Context

- **Main findings:** A direct association between social vulnerability and COVID-19 infection was observed in four states and the Federal Capital Territory in Nigeria, particularly later in the pandemic when COVID-19 restrictive measures have been relaxed.
- **Added knowledge:** Our findings add to the limited literature describing this association in Africa, particularly in Nigeria, and using a more robust and representative large household COVID-19 seroprevalence survey.
- **Global health impact for policy and action:** Our findings suggest the need for developing and using social vulnerability indexes in Africa to inform decision and policy making when prioritizing communities for resource allocation as part of emergency response and preparedness.

## Background

Socially vulnerable individuals in the community, including the poor, racial and ethnic minorities, children, elderly, and disabled, are more adversely affected during catastrophic events [1]. A common approach to estimating social vulnerability is by using a composite index, such as the social vulnerability index (SVI), which combines multiple factors corresponding to key social determinants of health [2]. This has the unique advantage of unifying multiple but related social factors into a single measure, thus enabling graded assessment of vulnerability on a quantitative scale as against the binary approach often used for individual factors [3].

SVI has been shown to be a strong predictor and a valuable indicator of disparities in health outcomes, including for cardiovascular disease, cancer, mental health, disability, and overall mortality [4–6]. Several studies have reported significant associations between SVI and morbidity and mortality due to COVID-19 infection [7–11]. However, a limited number of SVIs have been developed and used to explore the association with COVID-19 infection across Africa [12–16].

Utilizing a similar approach as employed by earlier developers, Lawal and Osayomi created and published, elsewhere [12], an SVI aimed at exploring key social determinants of health in relation to COVID-19 infection among the Nigerian population. We aimed to explore the association of this specific SVI with COVID-19 infection in Nigeria using a large household COVID-19 seroprevalence survey.

## Methods

We utilized data from a large population-based household COVID-19 seroprevalence survey conducted in Nigeria, the methods for which have been previously described [17]. In brief, the seroprevalence survey employed two-stage cluster sampling. All people in the household were eligible for inclusion, consenting participants completed an individual questionnaire, provided a blood sample for SARS-CoV-2 antibody testing, and had a nasal swab taken to test for active infection. The survey was conducted in two phases, phase one was conducted from September to November 2020 in Enugu, Gombe, and Nasarawa states. The second phase was conducted in June 2021 in Kano state and the Federal Capital Territory (FCT). Weighted COVID-19 seroprevalence estimates accounting for non-response, non-coverage, and survey design were generated and merged with Lawal and Osayomi et al. COVID-19-specific SVI [12] at the Local Government Areas (LGA) level, Nigeria’s third tier of government, below state and federal governments. The COVID-specific SVI incorporates known COVID-19 risk factors such as poverty, access to improved sanitation and potable water, proximity to airports and seaports, urbanicity, intensity of economic activity, and population density [12]. Linear regression models were used to estimate the effect of the SVI on COVID-19 seroprevalence. The effect of SVI was evaluated as both a continuous value and quintiles to evaluate a dose–response effect. Preliminary models were adjusted by phase, which was indirectly adjusted for time period. Final inferential models were stratified by the survey phase. Maps to spatially represent the distribution of COVID-19 seroprevalence and SVI were created using ArcGIS Pro.

## Results

A total of 75 LGAs were sampled across the four states and the FCT. From September to November 2020, COVID-19 seroprevalence ranged from 3.7% to 36.6% in Gombe, Enugu, and Nasarawa at the LGA-level. At the state-level, Gombe had the lowest seroprevalence [9.3% (95% Confidence Interval (CI) 7.0%–11.5%)], while Enugu had the highest at 25.2% (95% CI 21.8%–28.6%) (Figure 1a). Later into the pandemic (June 2021), seroprevalence ranged from 26.2% to 59.9% in the FCT and Kano at the LGA-level. At the state-level, the seroprevalence of the FCT and Kano was 40.3% (95% CI 34.7%–45.9%) and 42.6% (95% CI 39.4%–45.8%), respectively (Figure 1b).

**Figure 1.**
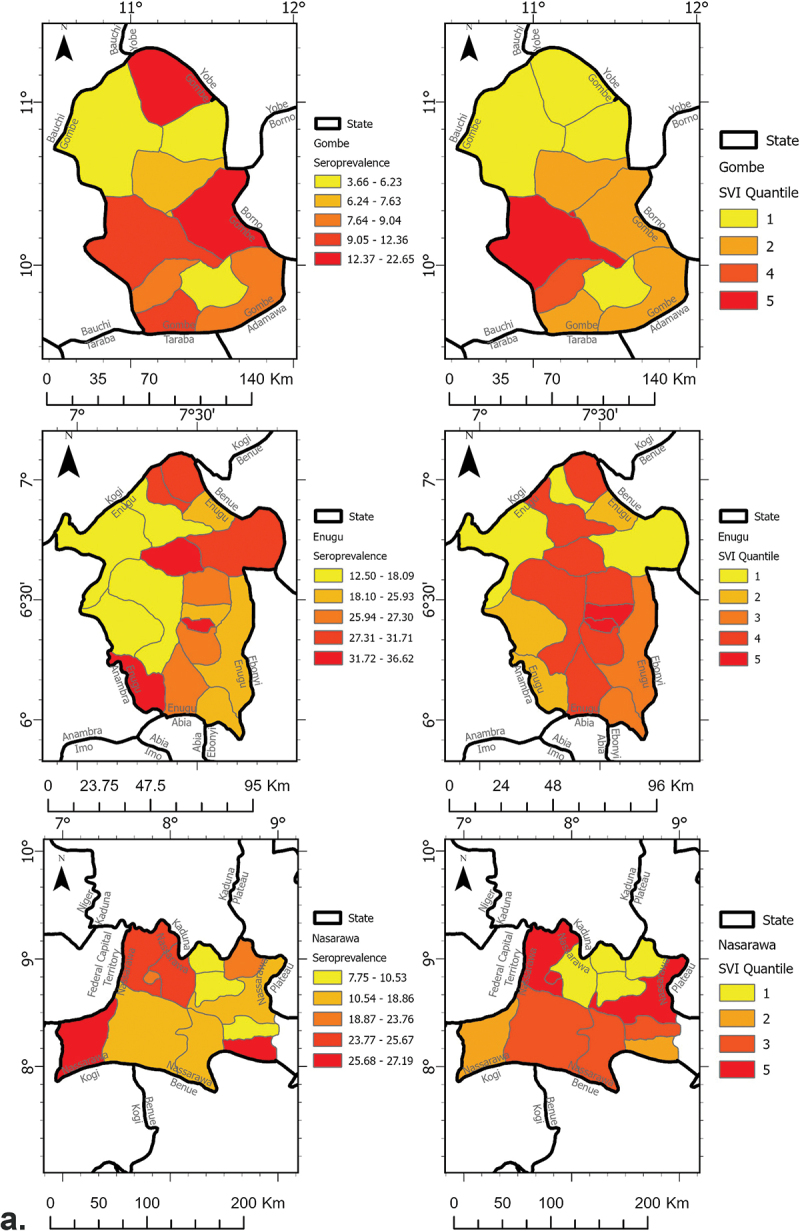
Maps depicting the spatial distribution of COVID-19 seroprevalence and SVI by state and survey phase, (a) phase 1 (Gombe, Enugu, Nasarawa), and (b) phase 2 (Kano, the FCT).

**Figure 1.**
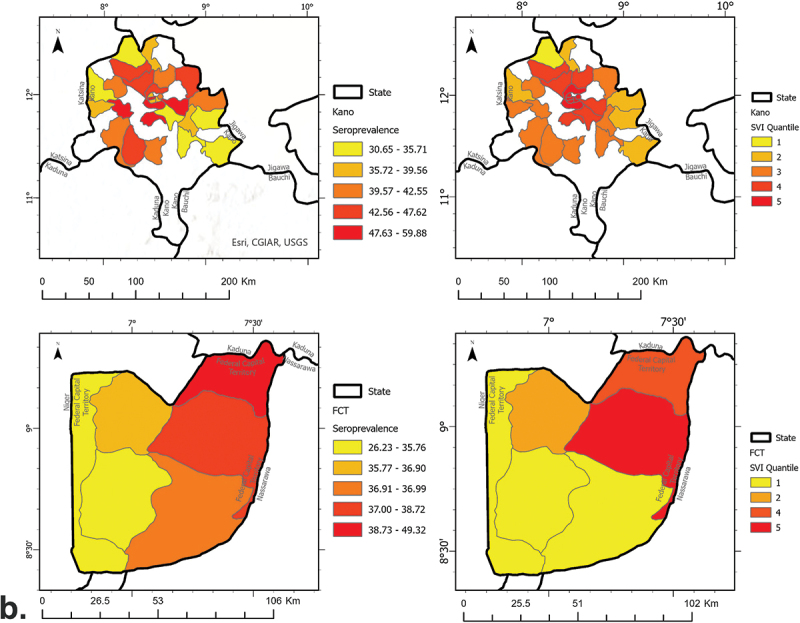
(Continued).

Across these four states and the FTC, the SVI values ranged from 2.8 to 5.8 at the LGA-level. The FCT had the highest average SVI value [3.6 (standard deviation (SD)) = 1.1], while Enugu and Nasarawa had the lowest SVI average at 3.3 (SD = 0.2 and 0.3, respectively) at the LGA-level (Figure 1a and b).

**Figure 2.**
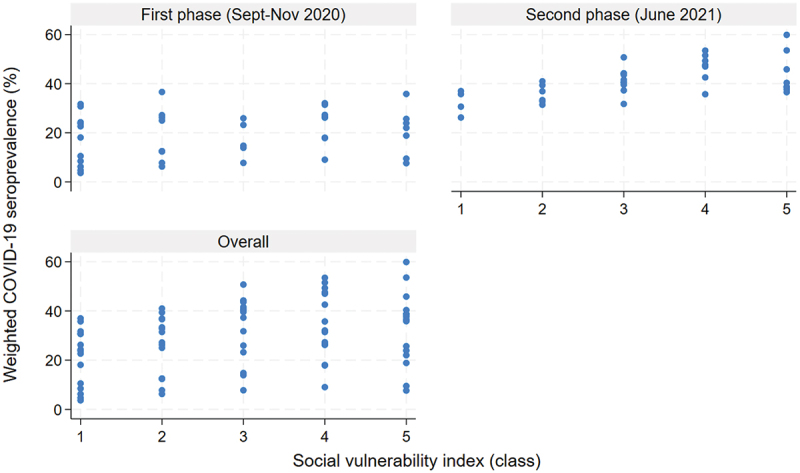
Relationship between COVID-19 seroprevalence and SVI by phase of survey (top) and overall (below).

Table 1 displays the regression analysis coefficients. Some evidence of an association between increasing SVI and increasing seroprevalence [β = 1.61 (95% CI 2.82, 6.05)] was observed between the continuous SVI value and COVID-19 seroprevalence after adjusting for the survey phase. If the LGA’s SVI increased by 1 unit, the COVID-19 seroprevalence increased by 1.61%, on average. When SVI was analyzed as quintiles, classes 4 and 5 (the most vulnerable groups) were associated with higher COVID-19 seroprevalence [class 4: β = 9.21 (95% CI 3.28, 15.15); class 5: β = 6.42 (95% CI 0.45, 12.40)] when compared to class 1 (the least vulnerable group) (Figure 2). Compared to class 1 (the least vulnerable group), COVID-19 seroprevalence was, on average, 9.21% and 6.42% higher in classes 4 and 5 LGAs, respectively, after adjustment by phase of the survey. These findings suggested higher COVID-19 seroprevalence in more socially vulnerable LGAs.

**Table 1.** Association between COVID-19 seroprevalence and SVI in Nigeria.

Social vulnerability index (SVI)	Overall weighted LGA-level COVID-19 seroprevalence *N* = 75			Weighted LGA-level COVID-19 seroprevalence					
				First phase (Sept–Nov 2020) *n* = 40			Second phase (June 2021) *n* = 35		
	Coefficient*	95% CI	*p*	Coefficient	95% CI	*p*	Coefficient	95% CI	*p*
SVI value (continuous)	1.61	−2.82, 6.05	0.470	−1.28	−10.16, 7.61	0.773	2.96	−1.73, 7.64	0.208
SVI class									
Class 1 (the least vulnerable)	Ref.			Ref.			Ref.		
Class 2	1.66	−4.24, 7.57	0.576	1.69	−7.03,10.41	0.696	3.40	−4.57, 11.37	0.390
Class 3	3.96	−2.13, 10.04	0.199	0.30	−10.16, 10.77	0.953	8.99	1.68, 16.30	0.018
Class 4	9.21	3.28, 15.15	0.003	6.73	−2.28, 15.75	0.138	14.34	6.59, 22.08	0.001
Class 5 (the most vulnerable)	6.42	0.45, 12.40	0.036	3.68	−5.70, 13.06	0.431	11.50	3.94, 19.07	0.004

*Linear regression models adjusted by survey phase.

After stratifying by survey phase, the association between the SVI quantiles and COVID-19 seroprevalence was apparent only during the second phase [class 5 vs. 1: β = 11.50 (95% CI 3.94, 19.07)]. Compared to class 1 (the least vulnerable group), the COVID-19 seroprevalence was, on average, 11.50% higher in class 5 LGAs. Furthermore, the strength of the association increased during this second phase compared to the combined-phase model [class 5, second phase β = 11.50 (95% CI 3.94, 19.07); class 5 combined-phase model: β = 6.42 (95% CI 0.45, 12.40)] (Table 1, Figure 2). Additionally, a dose–response relationship between COVID-seroprevalence and SVI quintiles was observed during the second phase of the survey. In the second-phase strata, the magnitude of the average seroprevalence increase changed from 3.40% in class 2 to 8.99% and 14.34% in classes 3 and 4, respectively. In class 5, although still positively associated, the increase was slightly attenuated from 14.34% in class 3 to 11.50%. Although these observed associations were significant, the confidence intervals of all these coefficients overlapped (Table 1).

## Discussion

Our results demonstrate an association between social vulnerability and SARS-CoV-2 seroprevalence in four states and the FCT in Nigeria. The association between higher social vulnerability and higher SARS-CoV-2 prevalence was more apparent after the second wave of the epidemic (June 2021), when COVID-19 bans and lockdowns were relaxed [18]. A dose–response effect was also observed during this time. Our findings are consistent with the direct correlation described between this index and State-level reported confirmed COVID-19 cases in Nigeria by Lawal and Osayomi [12].

Although there are limited studies describing this association in Africa [12–16], several manuscripts have described similar findings in other settings. In the United States, Karaye et al. described a positive association between SVI and COVID-19 case counts at the county level [9]. Similarly, Acharya and Porwal reported a similar association at the state-level in India [10]. Furthermore, using data similar to the present study, Li et al. described COVID-19 seroprevalence increases with increasing social vulnerability in the United States [7]. Huang et al. also reported a similar dose–response relationship between COVID-19 transmissibility and different levels of a COVID-19 pandemic vulnerability index across the United States [19].

Our study is subject to several limitations. As an ecological study, we are unable to make individual-based inferences. Nevertheless, the SVI is meant to be a community-level index, not an individual-based one, measuring a community’s vulnerability to events such as outbreaks or natural disasters. Similarly, our analysis was also limited by the small number of states where the seroprevalence survey was conducted, which could have impacted the strength and shape of the dose–response effect observed. Nevertheless, even with less than national coverage, our study demonstrated that the most socially vulnerable areas had the highest burden of SARS-CoV-2 infection. Additionally, the regression model that was developed did not incorporate spatial structure. The spatial structure could affect the coefficient, standard error, and model efficiency; thus, subsequent studies should test its incorporation into the model. Further studies that include more states are warranted to confirm the observed association across other regions of the country.

Furthermore, COVID-19 vaccination in Nigeria had recently started when the second phase of this survey was conducted in Kano and the FCT. Vaccination coverage levels of 1.6% (95% CI 0.7%–2.4%) in Kano and 3.4% (95% CI 1.3%–5.4%) in the FCT were reported by the survey. Due to these low coverage levels, our study did not consider this variable in this analysis. However, further studies that evaluate the association between social vulnerability and COVID-19 vaccination in this setting could help identify whether social vulnerability impacts COVID-19 vaccination access and uptake [20].

In conclusion, our study found a direct association between social vulnerability and COVID-19 infection, particularly later in the pandemic when COVID-19 restrictive measures have been relaxed. The SVI has the potential to be a useful tool to effectively prioritize communities for resource allocation as part of emergency response and preparedness in Africa. Further development of SVIs, at least to the third-administrative level, across the African continent could play a critical role in supporting governments to better inform their public health and policy.
